# Evaluating the effectiveness of an evidence-based online training program for health professionals in eating disorders

**DOI:** 10.1186/s40337-019-0243-5

**Published:** 2019-05-13

**Authors:** Sarah Maguire, Ang Li, Michelle Cunich, Danielle Maloney

**Affiliations:** 1 InsideOut Institute, Charles Perkins Centre, The University of Sydney, Camperdown, Sydney, 2006 NSW Australia; 2 The Boden Institute of Obesity, Nutrition, Exercise & Eating Disorders, Faculty of Medicine and Health, Sydney Health Economics, Sydney Local Health District, Charles Perkins Centre, The University of Sydney, Camperdown, Sydney, 2006 NSW Australia

**Keywords:** Eating disorders, Evidence-based online training, Health professionals, Early identification, Treatment and prevention, Knowledge, Skills, Stigma

## Abstract

**Background:**

Early detection and treatment are essential to ensuring the best possible health outcomes for people with eating disorders (EDs). However, low diagnostic accuracy and a lack of specific ED training are common workforce challenges in Australia and internationally. Online learning provides a potential solution in facilitating the access to evidence-based training programs. The InsideOut Institute has developed the first online clinical training program in EDs to assist with educating health professionals in the identification, assessment, and management of EDs. The aim of the study is to evaluate the effectiveness of the online training program, *The Essentials*, in mitigating barriers to health professionals treating patients with EDs.

**Methods:**

Pre and post training questionnaires assessed participants’ attitudes, knowledge, and skills in relation to treating people with EDs. Demographic and work-related information (gender, discipline, work setting, practice length and remoteness) and participants’ ratings of the online learning experience and satisfaction on completion were collected. The Wilcoxon signed rank test was applied to test for changes in learning outcomes before and after completion of the program. A multivariate linear regression model was estimated for each of the learning outcomes with personal and work-related characteristics as covariates.

**Results:**

Among 1813 health professionals who registered for *The Essentials* program between 1 October 2013 and 31 July 2018, 1160 completed at least 80% of the five learning modules. There were significant improvements in confidence, knowledge, skills to treat EDs and a reduction in stigmatised beliefs among the 480 participants who completed both pre and post assessments. Results from the regression models suggest that psychologists, dieticians, and those working in rural areas were more willing to treat EDs after completing the program. Additionally, those working in hospitals and regional or rural areas experienced the largest improvement in confidence for treating patients with EDs.

**Conclusions:**

*The Essentials* program represents a new and effective way of meeting the educational needs of partaking health professionals working with ED patients. Greater investment in the development and testing of evidence-based online training programs for EDs may help to address some of the considerable workforce development challenges in EDs.

**Electronic supplementary material:**

The online version of this article (10.1186/s40337-019-0243-5) contains supplementary material, which is available to authorized users.

## Plain English summary

Early detection and treatment is essential to ensuring the best possible health outcomes for people with eating disorders (EDs). However, low diagnostic accuracy and a lack of specific ED training are common workforce challenges in Australia and internationally. Online learning provides a potential solution in facilitating the access to evidence-based training programs. The InsideOut Institute have developed the first online clinical training program in EDs to assist with educating health professionals in the identification, assessment, and management of EDs, called “*The Essentials*”. Between October 2013 and July 2018, 1813 health professionals registered for the program. There were significant improvements in confidence, knowledge, skills to treat EDs and a reduction in stigmatised beliefs. In particular, psychologists, dieticians, and those working in rural areas had larger increases in their willingness to treat EDs after completing the program; those working in hospitals and regional or rural areas experienced the largest improvement in confidence for treating patients with EDs; and health professionals in education indicated a significant increase in the level of knowledge. Reaching to a wide audience at a relatively low cost, *The Essentials* program represents an effective way of meeting the educational needs of partaking health professionals working with ED patients.

## Background

Eating disorders (EDs) are serious psychiatric illnesses with severe physical and psychosocial consequences [1, 2]. The core features of EDs include disturbance to feeding and eating and disturbance of body image, resulting in extreme eating and/or purging behaviours that can lead to severe underweight, or in some cases overweight [3]. EDs are increasingly recognised as an important cause of morbidity and mortality, especially among young adult women [4, 5], although cases in men are not at all uncommon as more recent recognition supports [6, 7]. Individuals with EDs have high rates of role impairment, medical comorbidity, all-cause mortality and suicide [8–11]. Among the ED diagnostic entities, anorexia nervosa (AN) has been shown to have the highest mortality rates of any psychiatric disorder [1, 12].

Early diagnosis and intervention has prognostic benefits in limiting the ED’s progression and improving physical and psychosocial outcomes [13, 14]. EDs are clinically challenging for mental health services due to the high medical risks brought on by the disorder [15]. It is commonly reported that health professionals from various clinical disciplines lack confidence and training in screening, diagnosing and treating people with EDs [16–18]. In a number of studies, health professionals report a lack of adequate skills to work with people with EDs, and frequently report a need for more training on how to assist people with these complex conditions [19–22]. In particular, poor mental health literacy among health professionals has inhibited the effective treatment and recovery process of people with EDs [23–25]. Health professionals with more knowledge were more likely to ensure the recognition of the symptoms, the uptake of follow-up appointments, and the provision of effective referral or treatment [24, 25].

Lack of training, experience, and inadequate educational resources have also been associated with negative attitudes of health professionals towards people presenting with an ED [15]. These negative or dismissive perceptions are likely related to the nature of the symptoms and blame-based stigmatisation [26–28]. People with EDs are more stigmatised than people with depression and obesity [29, 30], and their illness is often seen as self-inflicted or attention seeking [31, 32]. Improvement in the person’s symptoms and their therapist’s perceptions of EDs are highly correlated factors [33], and the negative attitudes of health professionals can have a significant effect on both treatment alliances and outcomes [25, 34, 35].

To address the shortage of ED training courses, particularly for rural clinicians, online learning programs present as a viable alternative, enabling skill development in the participant’s own time and location. Compared to conventional face-to-face training, online learning can provide knowledge and skills demanded among non-specialist health professionals for diagnosis, treatment, and recovery, at relatively low cost and without geographical constraints. Moreover, the technology used for teaching allows for the incorporation of essential components of interactivity and feedback into the program, optimising the effectiveness of the learning process and outcomes. Several studies have demonstrated positive impacts in relation to confidence, knowledge, skills, competence, clinical practice, and satisfaction following online training specifically for health professionals [36–40]. However, the effectiveness of health-provider trainings varies in relation to rigorousness in study design and training methods. For example, active and behaviourally-oriented trainings (e.g. role-play), multicomponent training packages, large training curriculums, and ongoing support (e.g. consultation) were found to be effective in producing positive outcomes [41, 42].

A comprehensively designed online learning program, *The Essentials*, was developed for all health professionals (i.e. psychologists, dietitian, doctors, nurses and other allied health staff) working in EDs by the InsideOut Institute (formerly the Centre for Eating Disorder and Dieting Disorders [CEDD]) in Australia. A preliminary study of 187 learning participants of the program in 2012–13 demonstrated that the program was an effective tool in increasing health professionals’ level of knowledge, skill and confidence to treat people with EDs [40]. The results also demonstrated that online training reduced health professionals’ personal bias towards people with EDs. The current study examines the effectiveness of the online learning program with a much larger sample size over a longer period. Between 1 October 2013 and 31 July 2018, 1813 health professionals from a wide range of disciplines and work settings participated in *The Essentials*.

*The Essentials* program provides comprehensive training in relation to the medical, psychological and dietetic management of patients with EDs. The course incorporates expert videos, role-pays and interactive exercises and quizzes; and teaches extensive skills and techniques for diagnosis, assessment, treatment, management and dealing with service setting issues across all major EDs (i.e. AN, Bulimia Nervosa (BN), Binge Eating Disorders (BED), and Eating Disorder Not Otherwise Specified (EDNO)). *The Essential* has the advantages of being highly interactive, including a very comprehensive curriculum, and covering a much wider range of topics than previous online training programs which were limited to one particular treatment or to just prevention. Compared to other web-based training programs in EDs [38, 43], it has also reached a more heterogeneous group of professionals across professional disciplines.

The aim of the study is to examine the effectiveness of *The Essentials* program as measured by improvements in the level of willingness, confidence, knowledge, skill, and attitudes among participants upon completion. To better understand how and for whom the program was most beneficial and best suited, heterogeneity in learning outcomes was assessed by professional discipline, work setting, and geographical location of employment using multivariate linear regression analysis. Data from the in-built post online learning satisfaction measures was used to evaluate useability and learning experience of the program. By assessing the learning outcomes of a large sample of health professionals from diverse disciplines and different geographical locations, this study provides empirical results on the effectiveness of a new comprehensive online program, *The Essentials*, for professional development of the partaking EDs health workforce.

## Methods

### The essentials program

The InsideOut Institute’s online training program *The Essentials* addresses the nature, identification, assessment and treatment of EDs. The program was advertised via online advertisements on professional body’s websites including social workers, psychologists, nurses, mental health nurses, general practitioners and rural and remote medicine; eating disorders organisation’s websites; general mental health websites; the InsideOut website; and email list services that mainly targeted Australian audiences and by nature of being online reached a small number of international interested registrants. Two state governments in Australia (New South Wales [NSW] and Victoria) additionally purchased registrations on the program for their public health staff, as have other major health providers in Australia (e.g. Headspace [https://headspace.org.au] and Ramsay Health Care [https://www.ramsayhealth.com]. Health professionals registered from Australia and with a small number of registrations coming from other countries (Argentina, China, Egypt, Fuji, Ireland, Malta, Malaysia, New Zealand, Singapore, South Africa, the United Arab Emirates, the United Kingdom, the United States, and Vietnam) progressively from 1 October 2013 to 31 July 2018, and had 3 months to complete *The Essentials* program.

This program was funded by, and developed in collaboration with NSW Health. Training modules and topics were designed and written by internationally renowned expert and novice (or newly active) panels of health professionals in the field inclusive of nurses, dietitians, psychologists, psychiatrists and general practitioners, and curriculum development was based on literature reviews and expert consensus building [40]. The program comprises five modules: Understanding EDs and Diagnosis, Assessment, Preparation for Treatment, Treatment Approaches, and Management. Each module takes approximately 3.5 h to complete, and combines text-based psycho-education, role-plays, interactive exercises and quizzes, as well as video footage of sufferers and their families. Each of the five learning modules contain a core curriculum, required to pass the quiz, an in-practice section with additional clinical tools and role-plays, and a resources section with key readings and resources.

### In-built evaluation

A variety of assessment domain was examined in the study. Participants completed a self-evaluation questionnaire before and after completion of *The Essentials* program using Likert scales to measure changes in learning outcomes. The questionnaire was designed to cover best practice in evaluating training programs including what knowledge, skills, and attitudes are necessary to achieve the desired behaviour change in participants attending training [44]. The questionnaire included questions about willingness to treat different types of EDs (1 = Not at all willing and 5 = Willing); confidence in treating different types of EDs (1 = Not at all confident and 5 = Confident); personal beliefs about people with an ED (1 = Strongly Disagree and 5 = Strongly agree); and contributing factors to the development of EDs (1 = Does not contribute at all and 7 = Contributes very much). There were also questions regarding knowledge about the medical, psychological and dietetic management of EDs and skills in identifying and treating patients with EDs (1 = Very low and 5 = Very high). The individual questions were provided in the Additional file 1. The scale reliability coefficient (Cronbach’s alpha) was 0.96 for willingness questions, 0.95 for confidence questions, 0.96 for knowledge questions, 0.95 for skill questions, 0.78 for personal belief questions, and 0.77 for stigmatisation questions. All scales had reasonably strong alpha coefficients, which indicated good internal consistency within each scale.

**Fig. 1:**
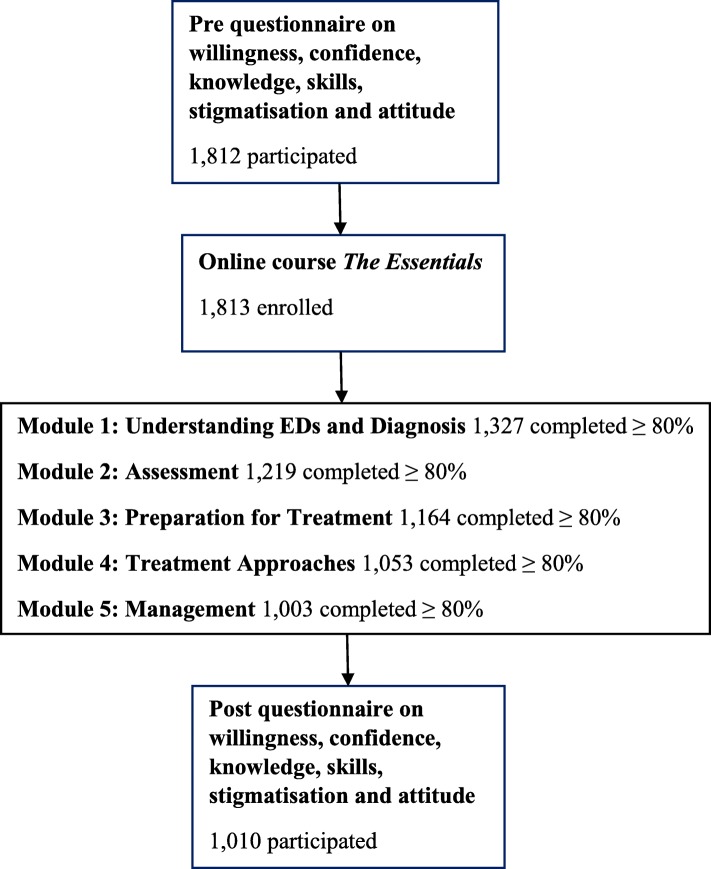
Flowchart of registration and participation of *The Essentials*

Additional items assessing demographic and work-related information including gender, age, Australian state/territory they reside in, primary professional discipline, work setting, length of time in practice and prior experience treating EDs were included at baseline. Participants also rated their experience of the online training program and satisfaction with the program. Participants when enrolling in the online program are asked to give consent to their information to be used for research purposes in a de-identified form. The process of registration and participation is illustrated in Fig. 1.

### Statistical analysis

Given the non-normal distribution of *The Essentials* questionnaire data, Wilcoxon signed rank test [45] was used to evaluate changes in willingness, confidence, knowledge, skills, and attitudes of health professionals in managing EDs before and after completing the program. The tests were performed on both unmatched (defined as participants who completed either the before or after questionnaire but not both) and matched participants (defined as those who completed both the pre and post questionnaires). The test for unmatched sample assessed the changes in learning outcomes for all observations recorded in the pre and post evaluations, while the test for matched sample ensures the comparison between pre and post outcomes for the same individuals. Ordinary Least Squares (OLS) regression analysis was used to examine the effect of work-related characteristics (i.e. discipline, work setting, years of practice, and employment remoteness) on the main learning outcomes (i.e. willingness, confidence, knowledge, skills, attitudes). All tests of significance were conducted at the 5% significance level. Family-wise error in multiple testing was addressed using Holm-Bonferroni method. All statistical analyses were undertaken in Stata version 14.

## Results

### Participation and completion rates

A total of 1813 health professionals registered for *The Essentials* from 1 October 2013 to 31 July 2018. Of these, 1160 completed at least 80% of the five learning modules and 1287 completed at least 80% of the first two modules (Understanding EDs and Diagnosis) which were considered to be the two most crucial components by the program’s developers and specialist clinicians. As is routinely observed, the completion rate decreased as the modules progressed [46, 47]. Using a completion rate of 80% as the benchmark, 74.1% completed the first module (Understanding EDs), 67.7% the second module (Assessment), 64.3% the third module (Preparation for Treatment), 58.5% the fourth module (Treatment Approaches), and 55.3% the fifth module (Management). Among the 1374 who completed at least one module, 78.3% completed the entire program (87.2% for the first module, 83.0% for the second module, 78.8% for the third module, 69.3% for the fourth module, and 73.0% for the last module).

### Characteristics of participants

Table 1 summarises the characteristics of participants who took up *The Essentials* program between 1 October 2013 and 31 July 2018. Participants were from a variety of disciplines, such as psychology (29.7%), nursing (21.7%), dietetics (15.4%) and social work (11.0%). There was also diversity in terms of where participants were primarily employed, ranging from community health centres (30.6%), hospitals (27.0%), private practice (15.6%), ED treatment services (7.5%), and education environment (3.9%). Almost a third of the sample (31.8%) had been in practice for less than 2 years, 41.1% for 3–10 years, and 27.1% for more than 10 years. Additionally, 52.5% of participants worked in metropolitan areas and 44.3% in regional or rural areas. The majority of participants were female (90.3%).

**Table 1:** Characteristics of participants, pre-engagement questionnaire (*N* = 1813)

	n	%
Gender		
Female	1637	90.29
Male	176	9.71
Residential location		
NSW	883	48.70
VIC	672	37.07
ACT	32	1.77
WA	19	1.05
NT	22	1.21
QLD	57	3.14
TAS	7	0.39
New Zealand	23	1.27
Other	98	5.41
Employment setting		
Private practice	283	15.61
ED treatment service	135	7.45
Community health centre	134	7.39
Community mental health centre	421	23.22
Hospital setting medical	212	11.69
Hospital setting psychiatric	278	15.33
Headspace	73	4.03
General practitioner (GP) clinic	26	1.43
Education/Teaching environment	70	3.86
Not currently employed	36	1.99
Other	145	8.00
Professional discipline		
Physician	12	0.66
Nurse	394	21.73
GP	38	2.10
Social worker	199	10.98
Psychiatrist	58	3.20
Psychologist	538	29.67
Dietician	279	15.39
Occupational therapist	83	4.58
School counsellor	14	0.77
Other	198	10.92
Length of practice		
< 2 years	577	31.83
3–5 years	412	22.72
6–10 years	333	18.37
11–15 years	173	9.54
16–20 years	125	6.89
20–30 years	125	6.89
> 30 years	67	3.70
Employment area		
Metropolitan	951	52.45
Regional	535	29.51
Rural	269	14.84
Not currently employed	58	3.20
EDs seen in practice		
AN	1387	76.50
BN	1068	58.90
BED	933	51.46
EDNOS	960	52.95
No ED patients	222	12.24
Inability to treat EDs		
Never	258	14.24
Rarely	404	22.30
Sometimes	844	46.58
Often	249	13.74
Always	57	3.15
Hours of training in ED screening and assessment		
0–5	1129	62.31
6–10	330	18.21
11–15	126	6.95
15–20	90	4.97
20–30	50	2.76
30+	87	4.80
Hours of training in ED intervention		
0–5	1177	64.96
6–10	297	16.39
11–15	110	6.07
15–20	69	3.81
20–30	51	2.81
30+	108	5.96

Notes: 1813 health professionals registered for The Essentials program from 1 October 2013 to 31 July 2018

Regarding the treatment provided to ED cases prior to completing *The Essentials* program, the majority of participants (*n* = 1591 or 87.8%) indicated that they saw patients with EDs in their main place of practice (Table 1). Of these, 83.2% of participants reported a caseload of 1–5 patients, with the majority treating cases of AN (85%), followed by BN (65.5% BN), EDNOS (59.4%), and BED (57.1%). Almost half of the sample (*n* = 844 or 46.6%) reported that they felt they were sometimes unable to treat patients presenting with an ED, and 306 (16.9%) indicated that they felt this “often” or “always”. Two thirds of participants stated that they had received only 0–5 h of training specifically on ED screening and assessment (62.3%), and 0–5 h of training specifically on ED intervention (65%).

### Improvements in willingness to treat patients with eating disorders

There was no significant increase in the willingness of matched participants to treat EDs after completing *The Essentials* program (z = − 0.889, *p* = 0.740) (Table 2). Failing to reject the equality of the willingness to treat patients with an ED may be explained by the sample consisting of program participants who already have a high inclination to assist patients with EDs (e.g. Table 2 shows a high pre evaluation score of willingness of 4.11).

**Table 2:** Pre and post evaluation of learning outcomes

	Pre-mean [SD]	Post-mean [SD]	Effect size	Z score
Unmatched (Npre = 1812, Npost = 1010)				
Willingness	4.11 [1.08]	4.17 [0.92]	0.07	0.66 (*p* = 0.51)
Confidence	2.44 [1.00]	3.41 [0.82]	1.11	24.33 (*p* < 0.01)
Knowledge	2.67 [0.71]	3.73 [0.56]	1.70	33.88 (*p* < 0.01)
Skills	2.59 [0.73]	3.47 [0.64]	1.28	28.28 (*p* < 0.01)
Stigmatisation	1.63 [0.54]	1.45 [0.50]	−0.33	−9.29 (*p* < 0.01)
Attitudes	1.04 [0.54]	1.11 [0.57]	0.04	2.93 (*p* < 0.01)
Matched (Npre = 480, Npost = 480)				
Willingness	4.10 [1.05]	4.17 [0.94]	0.06	0.89 (*p* = 0.74)
Confidence	2.40 [0.97]	3.42 [0.81]	1.09	17.19 (*p* < 0.01)
Knowledge	2.67 [0.66]	3.76 [0.55]	1.74	18.75 (*p* < 0.01)
Skills	2.57 [0.71]	3.45 [0.65]	1.29	18.24 (*p* < 0.01)
Stigmatisation	1.57 [0.49]	1.40 [0.47]	−0.38	−8.94 (*p* < 0.01)
Attitudes	1.13 [0.53]	1.15 [0.56]	0.13	0.56 (*p* = 0.74)

Notes: The unmatched sample is comprised of all participants who completed the in-built pre questionnaire or post questionnaire; and the matched sample is comprised of participants who completed both the pre and post questionnaires and could be matched using unique person ID identifiers. The relevant sample size is indicated in parenthesis. Wilcoxon-Mann-Whitney test and Wilcoxon signed rank sum test were performed, respectively, for unmatched and matched data to test the differences in the pre and post ratings reported by the participants. The Cohen’s d effect size was calculated. A Z score in bold indicates a statistically significance change at the 5% significance level. *P*-values were adjusted for familywise error rates using Holm-Bonferroni method. Estimates were rounded to 2 decimal places

### Improvements in confidence towards treating patients with eating disorders

There was a statistically significant increase in the confidence of participants towards treating patients with EDs of approximately one point in the mean measure (on a scale of 0–5) (z = − 17.188, *p* < 0.001). Breaking this down by diagnostic category, this equates to an increase of 0.93 points for AN (z = − 16.475, *p* < 0.001), 1.06 points for BN (z = − 17.154, p < 0.001), 1.07 points BED (z = − 17.115, *p* < 0.001), and 1.04 points for EDNOS (z = − 17.156, *p* < 0.001) (Table 2).

### Improvements in current knowledge of eating disorders

Participants reported on various aspects of their knowledge about treating patients with EDs, ranging from knowledge of resources available and current best practices, to knowledge about development, recovery and relapse of EDs. There was a significant improvement in knowledge about EDs after completing *The Essentials* program, with the mean score on the knowledge measure increasing from 2.67 to 3.76 (on a scale of 0–5) – a shift from the “low” to “moderate” level to nearly “high” level (z = − 18.748, *p* < 0.001) (Table 2).

### Improvements in current skills for treating and managing patients with eating disorders

There is a statistically significant increase (z = − 18.238, *p* < 0.001) in participants’ (mean) level of skills for treating and managing patients with EDs between pre and post engagement with *The Essentials* program (Table 2).

### Reduction in stigmatised perceptions towards patients with eating disorders

There was a significant decrease in the mean score for stigmatised beliefs towards patients with eating disorders among participants after completing the program (z = 8.938, *p* < 0.001) (Table 2).

### Improvements in personal beliefs towards patients with eating disorders

The increase in personal beliefs about the factors contributing to the development of an ED was statistically significant for unmatched participants (z = 2.930, *p* < 0.001) but not for matched participants (z = 0.556, *p* = 0.740). This may reflect that participants with more positive perceptions towards EDs were more likely to complete both pre and post assessment.

### Changes in the reasons for not treating patients with eating disorders

Participants who reported that they felt they were unable to treat patients with EDs were asked about the possible reasons or circumstances contributing to this perception. A total of 345 matched participants stated these reasons before and after completing the training. Lack of skills and limited resources were the most common barriers. Nevertheless, participants reported a significant decrease in perceived lack of skills to treat patients with EDs after completing the program, from 58.5 to 41.45%. After completing the program, the proportion of participants who indicated that treating EDs was too time consuming also dropped from 5.22 to 3.19%. In comparison, the number of participants who stated resources not in place for adequately treating EDs increased slightly from 35.65 to 40.87%.

### Evaluation of *The Essentials* online training program

*The Essentials* online learning program received highly positive evaluations from participants. Of the 1010 participants who completed the post training evaluation, 96.8% indicated their expectations of *The Essentials* program were met, 90.5% indicated they were satisfied with the program, and 96.4% indicated that the program met their learning needs well (Table 3). 99.4% of participants also indicated that the program was relevant or very relevant to their practices, and 94.4% indicated that their current practice has improved through completion of the program. Participants found the written text (97.6%), the videos (94.0%), the activities (93.1%), the quizzes (92.0%), the role-plays (90.7%), and the required readings (84.8%) useful components designed for ED training. 99.1% of participants stated that they would recommend *The Essentials* program to other clinicians in EDs.

**Table 3:** Post evaluation of *The Essentials* program (*N* = 1010)

	n	%
Expectation met		
Yes	978	96.83
No	32	3.17
Relevance to practice		
Entirely relevant	585	61.77
Partially relevant	356	37.59
Not relevant	6	0.63
Satisfaction with the program		
Very satisfied	414	43.72
Satisfied	443	46.78
Neither satisfied or unsatisfied	33	14.46
Unsatisfied	1	0.11
Very unsatisfied	56	5.91
Meeting learning needs		
Very well	463	48.89
Reasonably well	450	47.52
Neither	21	2.22
Not that much	11	1.16
Not at all	2	0.21
Improvement in current practice		
Definitely	562	59.35
Somewhat	332	35.06
Neither	23	2.43
Not that much	27	2.85
Not at all	3	0.32
Usefulness of the components		
Written text useful	816	97.61
Required reading useful	709	84.80
Videos useful	786	94.02
Activities useful	778	93.07
Quizzes useful	769	91.99
Role plays useful	758	90.67
Recommending to other clinicians		
Yes	807	99.14
No	7	0.86

Notes: 1010 participants completed the online training program evaluation. For usefulness of the components, the component was defined as useful if participants indicated “very useful” or “somewhat useful”

### Results from the regression models for learning outcomes

Table 4 presents estimates from the separate regression models for the six learning outcomes (i.e. willingness to treat, confidence, knowledge, skills, stigmatisation, and attitudes). Linear OLS regressions were used in the subgroup analysis to investigate the key factors associated with changes in learning outcomes across participant characteristics. The covariates in the model were professional discipline, work setting, years of practising as a general health professional, and a remoteness measure for location of main workplace. The results show that dietitians and psychologists, on average, experienced greater improvements in their willingness to treat patients with EDs by 0.35 and 0.23 points, respectively (on a scale of 0–5), compared to General Practitioners (GPs), social workers, physicians, occupational therapists, and other disciplines, controlling for personal and work-related characteristics. Negative attitudes towards caring for patients with EDs (stigmatisation) were shown to decrease most significantly among dietitians after completing the program, relative to other professional disciplines.

**Table 4:** Learning outcomes heterogeneity

Coef. (95% CI)	Willingness	Confidence	Knowledge	Skill	Stigmatisation	Attitude
Professional discipline						
Nurse	−0.072	−0.069	−0.174	0.097	−0.129	−0.032
	(−0.335, 0.191)	(− 0.323, 0.185)	(− 0.375, 0.027)	(0.098, 0.293)	(− 0.280, 0.021)	(− 0.191, 0.128)
Psychologist	**0.227**	− 0.038	− 0.057	0.019	− 0.038	− 0.059
	(0.026, 0.427)	(−0.232, 0.155)	(− 0.211, 0.096)	(− 0.130, 0.168)	(− 0.152, 0.077)	(−0.181, 0.062)
Dietician	**0.349**	0.115	0.015	0.028	**−0.139**	−0.025
	(0.116, 0.582)	(−0.110, 0.340)	(−0.163, 0.193)	(− 0.145, 0.201)	(− 0.272, − 0.006)	(−0.166, 0.116)
Work setting						
Community	0.038	0.150	−0.037	0.116	0.054	−0.078
	(−0.161, 0.237)	(−0.042, 0.342)	(− 0.189, 0.115)	(− 0.032, 0.263)	(−0.060, 0.167)	(− 0.199, 0.042)
Hospital	0.114	**0.284**	0.041	0.080	0.022	−0.102
	(−0.104, 0.331)	0.074, 0.493)	(−0.125, 0.207)	(−0.081, 0.241)	(− 0.102, 0.146)	(− 0.233, 0.030)
Education	0.008	0.415	**0.359**	0.262	0.096	−0.094
	(−0.458, 0.473)	(−0.034,0.864)	(0.003, 0.714)	(−0.084, 0.607)	(− 0.169, 0.363)	(− 0.376, 0.188)
Years of practice						
< 2 years	− 0.007	0.153	0.019	0.037	−0.038	−0.050
	(−0.120, 0.187)	(−0.034, 0.339)	(− 0.128, 0.167)	(− 0.107, 0.181)	(−0.148, 0.073)	(− 0.167, 0.067)
3–5 years	−0.023	0.050	0.025	−0.027	0.026	0.003
	(−0.227, 0.181)	(−0.147, 0.246)	(− 0.131, 0.180)	(− 0.178, 0.125)	(−0.091, 0.142)	(− 0.120, 0.127)
Work remoteness						
Regional area	0.100	**0.268**	0.111	0.107	−0.041	−0.046
	(−0.085, 0.286)	(0.089, 0.446)	(−0.030, 0.253)	(−0.031, 0.244)	(− 0.147, 0.065)	(− 0.158, 0.066)
Rural area	**0.402**	**0.688**	0.173	**0.263**	−0.028	0.050
	(0.143, 0.660)	(0.439, 0.938)	(−0.025, 0.370)	(0.072, 0.455)	(−0.175, 0.120)	(−0.106, 0.207)
Not currently employed	0.180	0.262	−0.124	−0.164	− 0.177	−0.064
	(−0.261,0.622)	(−0.164, 0.688)	(− 0.461, 0.213)	(− 0.502, 0.173)	(−0.429, 0.076)	(− 0.339, 0.212)
R^2^	0.07	0.12	0.04	0.06	0.05	0.03

Notes: Regressions also controlled for gender and location. Estimated coefficients and 95% confidence intervals are reported. Coefficients in bold indicate the covariate had a significant effect on the learning outcome at the 5% level. Covariates were gender, residential location, professional discipline, work setting, employment remoteness, and years of practice. Categorical variables were dummy coded. The base category for professional discipline was GP/social worker/physician/occupational therapist/others. The base category for work setting was private practice/ED treatment service/Headspace/GP clinic/others. The base category for years of practice was above 5 years. The base category for work remoteness is metropolitan area

Across work settings, participants from medical and psychiatric hospital settings experienced a significantly larger increase in their level of confidence to manage patients with EDs, relative to participants in private practice, GP clinics, and other settings. Allied health professionals working mainly in the education environment experienced an increase in their knowledge regarding the management of people with EDs of 0.28 points (on a scale of 0–5), which was larger than the knowledge learning outcomes achieved by health professionals in other work settings.

Participants working in rural areas achieved a larger (and significant) increase in self-evaluated willingness to treat patients with EDs, compared to those in metropolitan areas. Also notable is that participants in both regional and rural areas achieved a larger (significant) increase in confidence to treat patients with EDs, compared to those in metropolitan areas. These findings suggest that online training programs, such as *The Essentials* program, may provide a potential solution for the urgent need for professional development of mental health service providers in relation to EDs in rural and remote settings. This can be of particular significance in the Australian context where rural and regional areas are generally also under-serviced [40, 48].

## Discussion

This study evaluated the effectiveness of a series of online learning modules in *The Essentials* program for EDs that provides clinical education to health professionals working in this clinical area. Between October 2013 and July 2018, 1813 participants received comprehensive training in the medical, psychological and dietetic management of patients with EDs; 1812 completed the built-in pre evaluation; and 1010 completed the built-in post evaluation. The results of the study suggest that participation in the online program was associated with increases in confidence, knowledge, skills, and attitudes, with higher means and lower variations in evaluation scores upon completion of the program. The feedback from the participants was also very positive regarding the layout and content of the program.

*The Essentials* program targeted a wide range of health professionals and had a significant impact on health education. Consistent with previous studies, the online training program was shown to be a cost-effective method of knowledge and skill building for mental health providers [37, 42]; in addition, there were also substantial increases in their confidence and positive attitudes towards treating patients with EDs. Given that early diagnosis and management of EDs is crucial for achieving better and more enduring patient outcomes [13, 14], addressing the lack of confidence, knowledge, and training among health professionals is needed to assist them in providing the best quality care. A comprehensively designed online learning program that is easily accessible (and using any platform) can thus be an effective method to enhance the confidence, knowledge, and skills of health professionals working with EDs.

In particular, after having completed *The Essentials* program, dietitians and psychologists had larger increases in their willingness to treat patients with EDs than health professionals working in other disciplines; health professionals working in hospital settings showed a larger increase in their confidence to manage ED patients compared to those from private or community settings; and health professionals in education and teaching indicated an increased level of knowledge that was much larger than that reported by those in other settings. Importantly, the study also suggests that *The Essentials* program was successful in disseminating EDs diagnosis and treatment training to a broad audience, especially for healthcare professionals in geographically remote areas.

The current study has some limitations. First, it lacks a control group or randomisation, and therefore the effectiveness of the current online learning modules relative to other training methods was not evaluated. However, the results from the study will be used to assist with the design and implementation of a randomised controlled trial involving *The Essentials* and other training programs. Second, there was no long-term follow-up, which meant that it was not possible to assess how long the online learning outcomes were sustained, and whether the self-reported changes in learning outcomes were effectively translated into changes in clinical practice. A related issue is that the learning outcomes were subjectively evaluated by the participants themselves. Post training tests designed to objectively examine the knowledge of participants may provide additional information on the effectiveness of the program. Third, collecting more information on the sociodemographic characteristics of completers and non-completers would be informative to develop strategies to better engage participants. Moreover, completion and participation can be further improved to best realise the potential of online trainings. As one of the key challenges for e-learning setting, the reach of the online learning program should be further expanded to promote the participation of more health professionals in EDs.

## Conclusion

This study provides empirical evidence that an online learning program can be an effective method to support the professional development of healthcare providers working in the area of EDs, especially the health workforce in remote geographical regions. Participation in *The Essentials* online training program was associated with an increase in the inclination, confidence, knowledge, skills, and positive attitudes of health professionals in the management of patients with EDs. Reaching to a wide audience at a relatively low cost, online learning programs for EDs such as *The Essentials* are a promising training method, enabling the distribution of learning materials to promote mental health literacy among health professionals and, in turn, improve the diagnosis and treatment of patients with EDs in the health system.

## Additional file

Additional file 1: Eating disorder online learning evaluation questionnaire regarding attitudes, knowledge and skills. (DOCX 24 kb)

## Abbreviations

AN: Anorexia Nervosa
BED: Binge Eating Disorders
BN: Bulimia Nervosa
CEDD: Centre for Eating Disorder and Dieting Disorders
EDNOS: Eating Disorder Not Otherwise Specified
EDs: Eating disorders
GP: General Practitioners
NSW: New South Wales
OLS: Ordinary Least Squares
