# Supplementary material for: Evaluating the effectiveness of an evidence-based online training program for health professionals in eating disorders
Source: J Eat Disord. 2019 May 13;7:14. doi: 10.1186/s40337-019-0243-5 (PMC6513519; doi:10.1186/s40337-019-0243-5)
Supplement: Supplementary file 1 — Eating disorder online learning evaluation questionnaire regarding attitudes, knowledge and skills. (DOCX 24 kb) [file 40337_2019_243_MOESM1_ESM.docx]

# **Additional file 1**

**Evaluation questions regarding attitudes, knowledge and skills**

**Please state your *willingness* to treat each of the following eating disorders:**

|  | Not at all willing  1 | 2 | 3 | 4 | Willing  5 |
| --- | --- | --- | --- | --- | --- |
| Anorexia Nervosa |  |  |  |  |  |
| Bulimia Nervosa |  |  |  |  |  |
| Binge-Eating Disorder |  |  |  |  |  |
| EDNOS |  |  |  |  |  |

**Please select your level of *confidence* in treating each of the following eating disorders:**

|  | Not at all confident  1 | 2 | 3 | 4 | Confident  5 |
| --- | --- | --- | --- | --- | --- |
| Anorexia Nervosa |  |  |  |  |  |
| Bulimia Nervosa |  |  |  |  |  |
| Binge-Eating Disorder |  |  |  |  |  |
| EDNOS |  |  |  |  |  |

**Please rate your current *knowledge* regarding each of the following items with the following scale:**

|  | Very low (very uninformed) | Low | Moderate | High | Very high (very informed) |
| --- | --- | --- | --- | --- | --- |
| The prevalence rates of eating disorders in your country |  |  |  |  |  |
| The local resources available to patients struggling with an eating disorder |  |  |  |  |  |
| The current best practices for working with eating disorders |  |  |  |  |  |
| The evidence based treatments available for eating disorders |  |  |  |  |  |
| The other conditions that often co-occur with eating disorders |  |  |  |  |  |
| The most effective ways to talk about weight management and weigh-ins with patients struggling with an eating disorder |  |  |  |  |  |
| The common presenting complaints of patients struggling with an eating disorder |  |  |  |  |  |
| The risk factors associated with the development of an eating disorder |  |  |  |  |  |
| The indicators of recovery for patients with eating disorders |  |  |  |  |  |
| The factors contributing to relapse in patients with eating disorders |  |  |  |  |  |

**Please rate your current *skill* level regarding each of the following items with the following scale:**

|  | Very low | Low | Moderate | High | Very high |
| --- | --- | --- | --- | --- | --- |
| Asking a patient about their eating habits, weight, and body image without unduly raising their defenses |  |  |  |  |  |
| Talking with a patient about their eating disorder |  |  |  |  |  |
| Making appropriate referrals to eating disorder specialists |  |  |  |  |  |
| Handling a situation where you believe a patient has an eating disorder, but they are denying it or minimizing the impact on their physical and emotional functioning/well-being |  |  |  |  |  |
| Handling a patients' disclosure that they have an eating disorder |  |  |  |  |  |
| Conducting an eating disorder screening |  |  |  |  |  |
| Targeting the symptoms of the eating disorder to bring about symptom reduction |  |  |  |  |  |
| Delivering evidenced based interventions for the eating disorders |  |  |  |  |  |
| Taking a patient suffering with an eating disorder through treatment from diagnosis to recovery |  |  |  |  |  |

**Please indicate your level of agreement with the following statements about individuals with an eating disorder.**

|  | Strongly Disagree | Disagree | Neutral | Agree | Strongly Agree |
| --- | --- | --- | --- | --- | --- |
| They are to blame for their condition |  |  |  |  |  |
| They are a danger to others |  |  |  |  |  |
| They are hard to talk to |  |  |  |  |  |
| They could pull themselves together if they wanted to |  |  |  |  |  |
| They would not improve with treatment |  |  |  |  |  |
| They are acting this way for attention |  |  |  |  |  |

**Please indicate the extent to which you believe the following factors contribute to the development of an eating disorder.**

|  | 7 (Contributes very much) | 6 | 5 | 4 | 3 | 2 | 1  (Does not contribute at all) |
| --- | --- | --- | --- | --- | --- | --- | --- |
| Poor living habits |  |  |  |  |  |  |  |
| Parenting |  |  |  |  |  |  |  |
| Biological factors |  |  |  |  |  |  |  |
| Lack of social support |  |  |  |  |  |  |  |
| Self-discipline |  |  |  |  |  |  |  |
| Society's thin ideal |  |  |  |  |  |  |  |
| Genetic factors |  |  |  |  |  |  |  |
| Psychological factors |  |  |  |  |  |  |  |
| Vanity |  |  |  |  |  |  |  |
